# Recent Advances in Forensic Anthropological Methods and Research

**DOI:** 10.3390/biology11060908

**Published:** 2022-06-13

**Authors:** Eugénia Cunha, Ann H. Ross

**Affiliations:** 1 Centre for Functional Ecology (CEF), Laboratory of Forensic Anthropology, Department of Life Sciences, University of Coimbra, 3000-456 Coimbra, Portugal; cunhae@ci.uc.pt; 2 National Institute of Legal Medicine and Forensic Sciences, 1169-201 Lisbon, Portugal; 3 Human Identification & Forensic Analysis Laboratory, Department of Biological Sciences, North Carolina State University, Raleigh, NC 276995, USA

This Special Issue, “Recent Advances in Forensic Anthropological Methods and Research”, with thirteen articles covers a wide range of highly diverse topics within forensic anthropology. Topics ranging from innovative approaches to critical reviews have received much attention, with more than thirteen thousand views during the past year. This is unequivocal proof of the interest in this Special Issue. Authors representing Europe, the United States, Australia, and South Africa embody the breadth of the present-day research being conducted in forensic anthropology.

In regard to estimating biological profiles (e.g., biological sex, age at death, population affinity, and stature), there are three articles focusing on age at death. One manuscript by Niel, Chaumoître, and Adalian [1] addresses bias due to altered growth trajectories in estimating juvenile aging in fetuses and infants. Two manuscripts discuss aging adults, considered to be the Achilles heel of forensic anthropology. A paper by Dias, Manco, Corte Real, and Cunha [2] proposes a blood–bone–tooth model using DNA methylation to predict age in forensic contexts. This paper presents an interesting alternative for aging the dead and the living, and brings new insights into the development of multitissue age prediction models as applied to blood, bone, and teeth. The third adult age estimation article by Navega, Costa, and Cunha [3] proposes a new method based on a multifactorial macroscopic analysis and deep random neural network models. Within the generic factors of identity (i.e., biological profile), the ever-polemic topic of population affinity is discussed and illustrated using geometric morphometric and spatial analysis methods within Latin America. Ross and Williams [4] argue that there is a benefit to and necessity of embracing studies that employ population structure models to better understand human variation and the historical factors that have influenced it.

Within the realm of individualizing factors, Butaric, Richman, and Garvin [5] discuss the potential factors that might affect the reliability of using frontal sinuses for personal identification. Their study investigates how slight deviations in orientations affect sinus size and outline shape, which could potentially impact identification.

New approaches are illustrated by the article by Procopio, Mein, Starace, Bonicelli, and Williams [6], which shows that bone proteomics is a well-founded resource with which to identify microbially driven versus extrinsically driven bone diagenesis. Another novel subject is the review by Marquez-Grant and colleagues on the effects of various drugs on the skeleton, including prescription and recreational drugs, that could affect forensic anthropological analyses [7]. Another new approach by McWhirter and colleagues describes how to accurately individualize skeletons from commingled remains using mesh-to-mesh value comparisons for pair matching skeletal elements [8].

A topic with increasing attention is forensic facial comparison, which is the subject of one paper by Bacci and coworkers that discusses relevant terminology, the validity as well as reliability of the Facial Identification Scientific Working Group’s list of morphological features, and proposes standards for CCTV equipment [9].

The need to know the attributions of each area of expertise in forensic anthropology is discussed by Passalacqua, Pilloud, and Congram [10], who call attention to ethical procedures and requisite qualifications. Furthermore, they emphasize the need to develop standards and best practice guidelines.

One of the main reasons why forensic anthropologists are called to testify in court is because of traumatic injuries to skeletal tissues. The article of de Boer, Berger, and Blau [11] discusses and examines the concept of ‘degree of force’ as well as why it is considered a pertinent issue in legal proceedings.

One of the big challenges in skeletal traumatic injuries interpretation is to perform discrimination among BFT and thermal-induced trauma. Keys and Ross [12] conducted an experiment that found that blunt force trauma signatures remained after burning. It concludes that there are distinct patterns attributed to thermal fractures and blunt force fractures.

Nonhuman skeletal remains continue to be part of the routine cases of forensic anthropologists. The Garvin team [13] assesses the utility of quantitative methods for distinguishing human from nonhuman remains and presents additional resources for species identification.

We can consider that we have accomplished our aims of presenting a wide array of methods and topics that are unquestionably relevant to the practice of forensic anthropology. The quality of expertise has to derive from modern and updated research.

*A teoria orienta, a experiência decide.*
